# Neutralization of Junín virus by single domain antibodies targeted against the nucleoprotein

**DOI:** 10.1038/s41598-018-29508-1

**Published:** 2018-07-30

**Authors:** Florencia Linero, Claudia Sepúlveda, Ioanna Christopoulou, Paco Hulpiau, Luis Scolaro, Xavier Saelens

**Affiliations:** 1 VIB Center for Medical Biotechnology, Ghent, B-9052 Belgium; 2 Department of Biomedical Molecular Biology, Ghent University, Ghent, B-9052 Belgium; 3 Laboratory of Virology, Faculty of Sciences, University of Buenos Aires, C1428EGA Caba, Argentina; 4 VIB Center for Inflammation Research, VIB, Ghent, B-9052 Belgium

## Abstract

The syndrome viral haemorrhagic fever (VHF) designates a broad range of diseases that are caused by different viruses including members of the family *Arenaviridae*. Prophylaxis for Argentine Haemorrhagic Fever (AHF), caused by the arenavirus Junín (JUNV), has been achieved by the use of a live attenuated vaccine, named Candid#1. The standard treatment of AHF is transfusion of convalescent human plasma. Our aim was to develop an alternative and safer treatment for AHF based on the use of virus-neutralizing single domain antibodies (VHHs). We describe the first reported VHHs directed against an arenavirus. These VHHs could neutralize Candid#1 by altering virion binding/fusion. Surprisingly, the neutralizing VHHs appeared to be specific for the viral nucleoprotein (N) that is not known to be involved in arenavirus entry. Candid#1 VHH-escape viruses had acquired a predicted N-glycosylation site in the surface glycoprotein GP1 that is present in highly pathogenic JUNV strains. Accordingly, the Candid#1-neutralizing VHHs could not neutralize pathogenic JUNV strains, but they could still bind to cells infected with a pathogenic strain or the escape mutant viruses. These results show that the attenuated strains of JUNV can be potently neutralized by nucleoprotein-specific VHHs.

## Introduction

Arenaviruses occur worldwide and can cause severe disease in humans. Members of the *Arenaviridae* family are enveloped viruses with a bi-partite RNA genome coding for four viral proteins. The viral nucleoprotein (N) and the glycoprotein precursor (GPC) are encoded by the small (S) genome segment, whereas the RNA-dependent RNA polymerase and the matrix protein (Z) are encoded by the large (L) segment. Both genome segments, S and L, use an ambisense coding strategy, with N and L oriented in negative sense and GPC and Z in positive sense relative to the genomic RNA, which is considered negative stranded. The *Arenaviridae* family is subdivided into two genera: Mammarenavirus, members of which infect mammals, and Reptarenavirus, members of which infect snakes^1–6^. Rodents that are chronically infected with these viruses represent the natural reservoirs of Mammarenaviruses. However, some members of the Mammarenaviruses sporadically cause zoonotic infections in humans, which may lead to life-threatening viral haemorrhagic fever (VHF) disease^7^.

Mammarenaviruses are classified in two groups based on their genetic and geographical distribution: Old World (OW) viruses are found in Europe and Africa and New World (NW) viruses in the Americas. At present, there are about 30 species of Mammarenaviruses described, 8 of which can cause severe VHFs in humans^7^. Humans can become infected with these viruses through direct contact with excreta of infected rodents or through inhalation of aerosols or dust particles that are contaminated with the virus. Person to person transmission is rare and has been reported for Lassa (LASV), Machupo (MACV) and Lujo (LUJV) viruses mostly during nosocomial care^8,9^.

VHFs caused by arenaviruses are acute diseases with considerable mortality rates. Symptoms are fever, malaise, headache, nausea, diarrhea, petechial hemorrhage of the soft tissues, lethargy, and irritability. However, in severe cases, patients can experience different degrees of bleeding, leukopenia, thrombocytopenia that are associated with a shock syndrome in the terminal stage^10,11^. The purine nucleoside analog ribavirin is the only off-label drug recommended for use in treating arenaviruses infections under emergency provisions^12,13^.

Argentine Hemorrhagic fever (AHF) is caused by the NW arenavirus JUNV, first isolated in Argentina from a human case in 1958^14,15^. The wild rodents *Calomys musculinus* and *Calomys laucha* are the main reservoirs of JUNV. The endemo-epidemic area of AHF has gradually expanded and currently comprises an area of 150.000 km^2^ and the population at risk is approximately 5 million people^16^. The incubation period for AHF is estimated to be between 7 and 14 days^17^. The health of infected patients may improve after one or two weeks, but approximately one-third of untreated cases become severely ill with bleeding tendencies and/or neurological signs. Worsening of these symptoms is often fatal and 15–30% of AHF patients succumb to the infection. At present the transfusion of human immune plasma into patients with a clinical diagnosis of AHF is the only standard specific treatment for this disease. Such transfusion is effective only when applied during the first week of infection^18^. Moreover, development of a late neurological syndrome in plasma treated patients, the scarcity of the immune plasma source and the risk of transfusion borne diseases are serious drawbacks of this treatment^19^.

Although prevention for AHF is possible through the use of a live attenuated vaccine, Candid#1^20,21^, there is a safety risk associated with the administration of live attenuated virus vaccines to children, pregnant women and immunocompromised persons^22,23^. To circumvent these risks, monoclonal antibody-based therapies are under consideration. A recent report describes the development of humanized monoclonal antibodies that can prevent disease and death in the JUNV guinea pig model^24^. On this line, antibodies that bind antigens via a single protein domain were discovered in 1990 in some members of the *Camelidae* family and are under active investigation for immune therapies^25^. These so called heavy chain-only antibodies lack a light chain which means that their antigen recognition is confined to a single variable domain, named as VHH. These VHHs, also known as ‘Nanobodies®’, are currently explored for biotechnological and therapeutic applications because of their small size, simple production and high affinity. We previously reported on the potential of recombinant VHHs to prevent and treat H5N1 influenza and human respiratory syncytial virus infection in experimental models^26–29^. In this work, we describe the selection and characterization of VHHs with potent *in vitro* antiviral activity against JUNV.

## Results

### Isolation and characterization of JUNV-specific single domain antibodies

**Figure 1:**
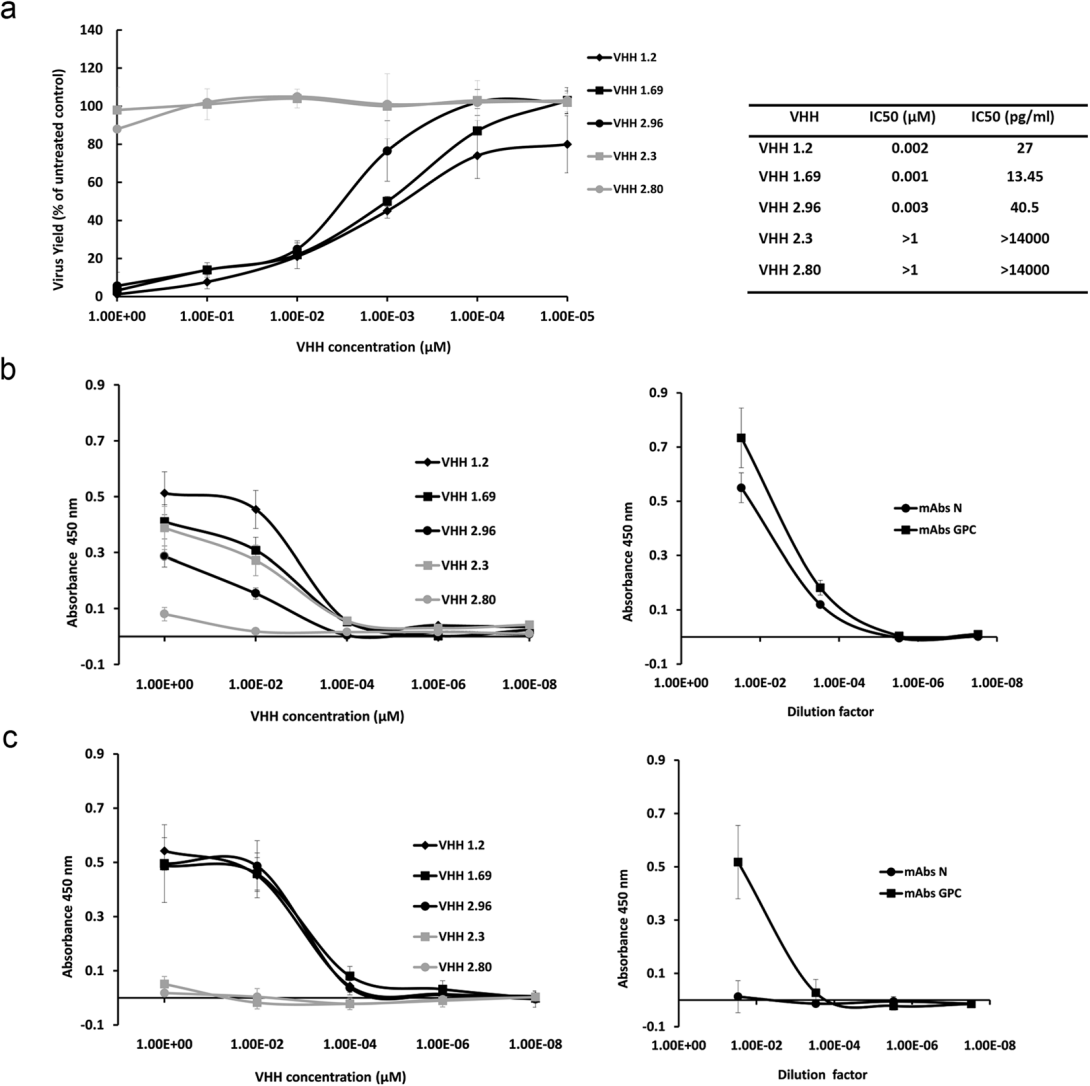
VHHs 1.2, 1.69 and 2.96 neutralize Candid#1 virions and bind to Candid#1 infected cells. (**a**) The 50% inhibitory concentration (IC_50_) of the purified VHHs was determined by infection of Vero cells with 50 PFU of Candid#1 that had been previously incubated with serial dilutions of the indicated purified His-tagged VHHs. At 3 days p.i. virus plaques were visualized and counted by immunostaining. Virus yield in the Y-axis is expressed as the percentage of plaques relative to the untreated control. The IC_50_ value (right) of the VHHs was calculated from three independent experiments (performed in duplicate) and error bars represent the SD of the mean. (**b**) The binding of the VHHs to infected cells was evaluated by ELISA in Vero cells infected with 50 PFU of Candid#1. At 72 h p.i, cells were fixed, permeabilized and afterwards incubated with serial dilutions of the indicated VHHs (left) or JUNV mAbs (right). (**c**) Alternatively, Candid#1 infected Vero cells were first incubated with serial dilutions of VHHs or JUNV mAbs and afterwards fixed, without prior permeabilization. VHHs binding curves are depicted in the left panels and N- and GPC-specific mAbs binding curves on the right. Results show the mean of three independent experiments and error bars represent the SD of the mean.

Candid#1-specific VHHs were selected from a phage display library that was generated after immunization of an alpaca with purified UV-inactivated Candid#1 virions. VHH candidates were selected by biopanning, analysis of their sequence diversity and their capacity to neutralize Candid#1 (Supplementary Figs 1 and 2a). Three VHHs, named 1.2, 1.69 and 2.96, were produced and purified from *E*. *coli* in mg amounts, and showed dose-dependent Candid#1 virus neutralizing activity with calculated IC_50_ concentrations in the low nM range (Fig. 1a). Additionally, VHH 2.3, which binds to Candid#1-infected cells but does not neutralize the virus, and VHH 2.80, which neither binds to nor neutralizes Candid#1 virus, were selected as controls (Fig. 1a). The predicted amino acid sequence of these selected VHHs revealed that the neutralizing VHHs 1.2, 1.69 and 2.96 have similar framework (FR) and complementarity determining regions (CDRs) (Supplementary Fig. 2b).

We next determined if the Candid#1 virus-binding VHHs could also bind to Candid#1 infected cells. VHH binding was assessed after fixation and permeabilization of Candid#1 infected Vero cell monolayers. All three neutralizing VHHs as well as the non-neutralizing VHH 2.3 bound to permeabilized, paraformaldehyde fixed infected cells whereas VHH 2.80 did not (Fig. 1b). As expected, JUNV N- and GPC-specific monoclonal antibodies (mAbs) recognized viral antigens in fixed and permeabilized Candid#1-infected cell monolayers (Fig. 1b, right panel).

Next, we evaluated the capacity of the VHHs to bind intact infected cells, *i*.*e*. without prior permeabilization. By doing so, JUNV antigens that are membrane-exposed could be recognized by the VHHs. Thus, the VHHs were allowed to react with Candid#1-infected cells before fixation and the permeabilization step was omitted. Under these conditions the three neutralizing VHHs still bound to infected cells, while VHH 2.3 (and VHH 2.80) did not (Fig. 1c). These results suggest that the neutralizing VHHs 1.2, 1.69 and 2.96 recognize a virus-associated surface exposed antigen on infected cells. As expected, anti-GPC mAbs specifically bound to infected cells that were not permeabilized in agreement with the surface-exposed nature of GPC (Fig. 1c, right panel). On the contrary, N-specific mAbs failed to bind under these conditions, in accordance with the expected cytoplasmic expression of the viral N (Fig. 1c, right panel).

**Figure 2:**
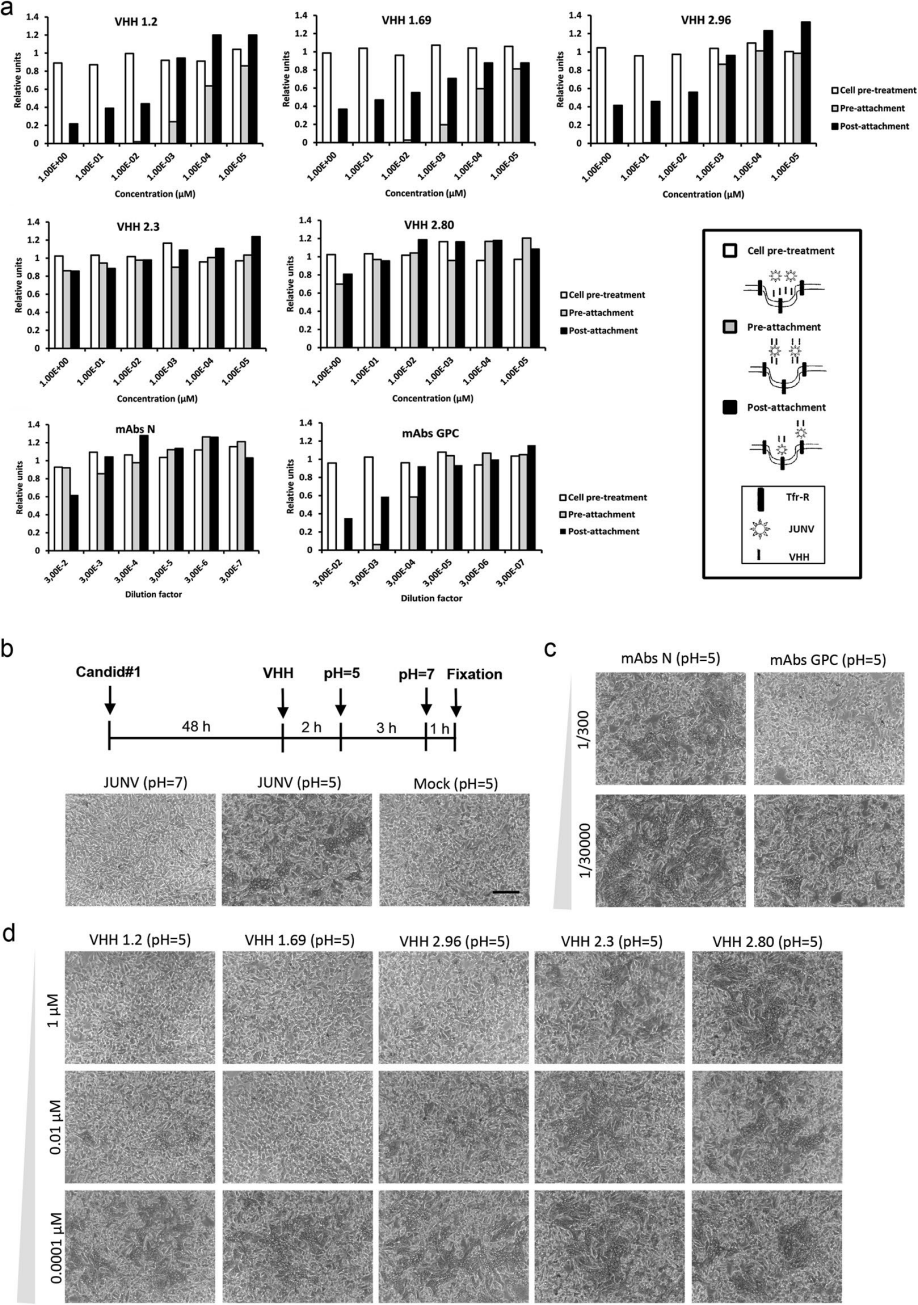
VHHs neutralize Candid#1 at a step following attachment. (**a**) Panels represent the results of the treatment with the indicated VHH or mAbs. The timing of the infection relative to the other treatments is schematically shown in the bottom right in which the transferrin receptor (Tfr-R), JUNV virions and the VHH, are schematized. For the cell pre-treatment assay, Vero cells were incubated with serial dilutions of VHHs for 1 h at 37 °C, then chilled on ice for 15 min and then incubated with 50 PFU of Candid#1 virus 1 h at 4 °C to allow virus attachment. After attachment, the cells were washed with cold medium and then shifted to 37 °C (white bars). For the pre-attachment assay 50 PFU of Candid#1 virus were mixed with serial dilutions of VHHs and incubated at 37 °C for 1 h prior to inoculation of Vero cells (gray bars). For the post-attachment assay Candid#1 virus virions were added to pre-chilled Vero cells, incubated for 1 h at 4 °C and afterwards incubated with 1/10 serial dilutions of VHHs during 1 h at 37 °C (black bars). At 3 days p.i. the extent of viral replication was quantified by ELISA. (**b**) Vero cells were mock infected or infected with Candid#1 at an MOI of 0.05 PFU/cell. At 48 h p.i. monolayers were mock treated or incubated for 2 h with serial dilutions of VHHs or JUNV-specific mAbs. Afterwards, syncytium formation was induced by incubation in acidic media (pH = 5) and the presence of multinucleated cells was evaluated by Giemsa staining (bright field pictures). (**c**) Syncytium formation in presence of serial dilutions of GPC and N- specific mAbs. (**d**) Syncytium formation in presence of serial dilutions of VHHs. Representative results from three independent experiments are shown. The scale bar in the bright field images represents 100 µm.

To obtain more insight into the neutralization process of the Candid#1-specific VHHs, we performed experiments in which the VHHs were added before or after virion attachment to Vero cells. No significant antiviral effect was observed when VHHs or control N- or GPC-specific mAbs were added to the cells before infection and subsequently washed away (Fig. 2a, white bars, cell pretreatment). This indicates that a host cell factor (*e*.*g*. transferrin receptor) involved in Candid#1 attachment or entry^30^ was not targeted by these antibodies. In contrast, the three neutralizing VHHs, like the GPC-specific mAbs, reduced infection dose-dependently when they were incubated together with the virus before attachment (Fig. 2a, gray bars, pre-attachment). Remarkably, incubation after virus attachment also resulted in inhibition of virus replication suggesting that the antiviral effect of the VHHs as well as GPC-specific antibodies is also operative after viral adsorption (Fig. 2a, black bars, post-attachment). The non-neutralizing VHHs 2.80 and 2.3, as well as the N-specific mAbs failed to suppress Candid#1 replication in any of the conditions evaluated (Fig. 2a). We can thus conclude that the neutralizing VHHs 1.2, 1.69 and 2.96 prevent infection by interfering with virion adsorption and/or uncoating, in a similar fashion as GPC-specific mAbs do.

Attachment of JUNV to its receptor, the transferrin 1 receptor, is followed by clathrin-mediated endocytosis, virion delivery into endosomes and a pH-dependent membrane fusion mediated by GPC^30,31^. To evaluate if the neutralizing VHHs could prevent membrane fusion, we performed a cell-cell fusion assays in Candid#1 infected Vero cells. Cells were infected with Candid#1 and 48 h after infection VHHs, N- or GPC-specific mAbs were added to the cells. Subsequently, the cells were kept at neutral pH or exposed to pH 5.0 for 3 h to trigger GPC-mediated syncytium formation. Finally, the pH of the medium was neutralized for 1 h and the cells were fixed and stained. The presence of multinucleated cells was analyzed by bright field microscopy (Fig. 2b). Syncytium formation in untreated Candid#1 infected cells was only observed after exposure of cells to pH 5 (Fig. 2b). As expected, GPC-specific mAbs but not N-specific mAbs, prevented pH-induced syncytium formation (Fig. 2c). All three neutralizing VHHs also prevented syncytium formation in a dose-dependent manner, while no effect was observed for VHH 2.3 and 2.80 (Fig. 2d). In summary, these results indicate that the neutralizing activity of VHHs 1.2, 1.69 and 2.96 correlates with their capacity to interfere with Candid#1-mediated syncytium formation.

### Candid#1-neutralizing VHHs are specific for the viral nucleoprotein

**Figure 3:**
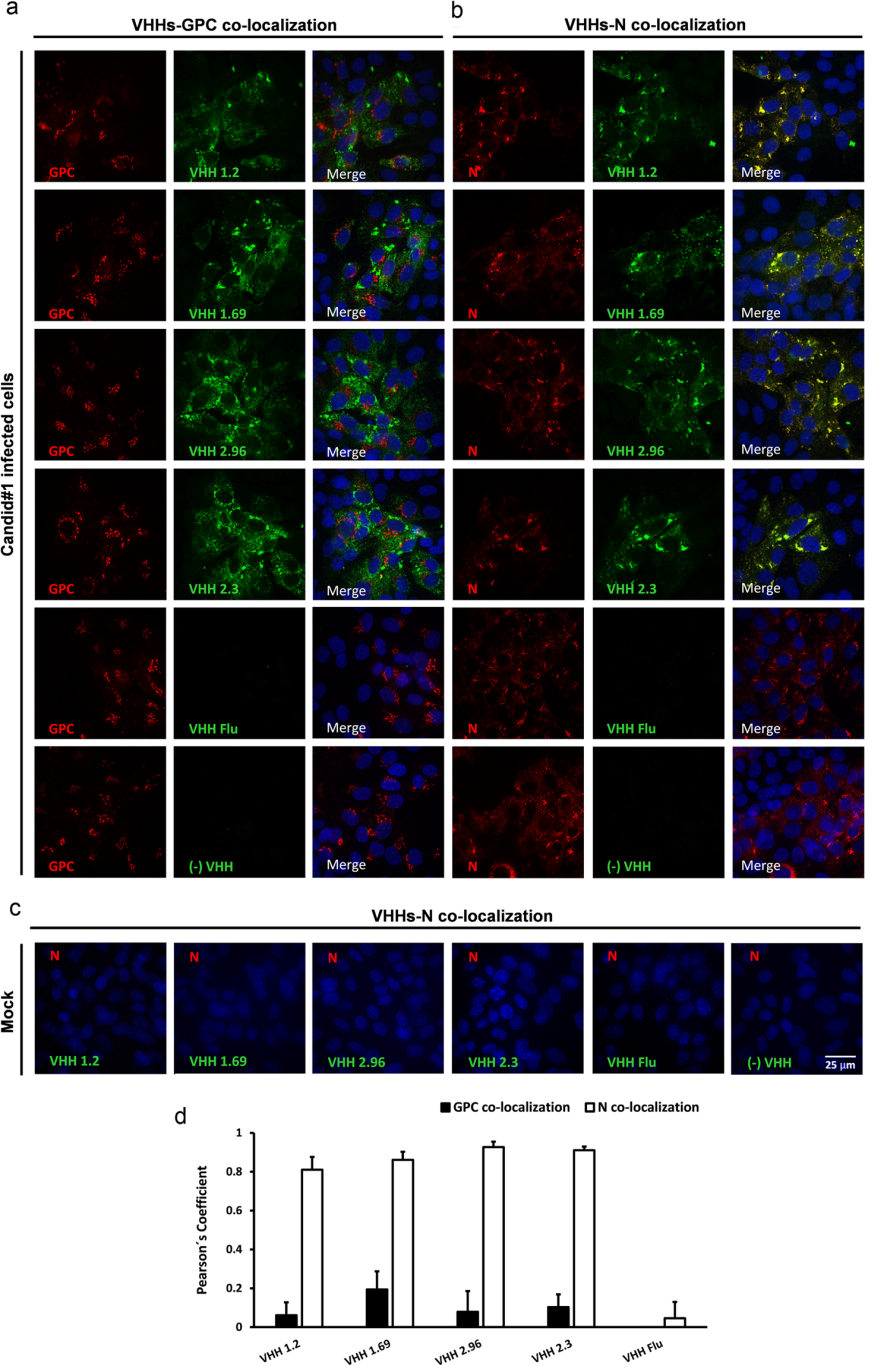
VHHs 1.2, 1.69, 2.96 and 2.3 colocalize with JUNV N-specific Mabs in fixed and permeabilized Candid#1 infected cells. Vero cells were mock infected or infected with 150 PFU of Candid#1. At 48 h p.i. cells were fixed, permeabilized and further incubated with 10 µM of VHHs followed by incubation with GPC- (**a**) or N-specific mAbs (**b**). Afterwards, monolayers were incubated with an anti-His antibody and finally incubated with the corresponding secondary antibodies. Anti-His antibody without the VHH was included as a negative control (-VHH) and incubation with a VHH against an influenza epitope (VHH Flu) was used as control as well. (**c**) In addition, immune-staining was performed in mock-infected cells with VHHs followed by incubation with N-specific mAbs. (**d**) Colocalization of the VHH-specific fluorescent signal with that of the GPC- an N-specific mAbs was determined in 50 cells that showed a positive signal with GPC- or N-specific mAbs. The Pearson’s correlation coefficient (P) was calculated using the Coste’s algorithm provided in the Volocity software package. The scale bar represents 25 µm.

To identify the viral antigen that is targeted by the VHHs, we performed co-localization assays with N- or GPC-specific mAbs in infected cells. In a first set of experiments, Vero cells were infected with Candid#1 virus, fixed, permeabilized and then incubated with the mAbs and the VHHs. GPC- and N-specific mAbs bound to the infected cells in a typical perinuclear dotted pattern for GPC and a granular cytoplasmic fluorescence pattern for N (Fig. 3a,b)^32,33^. Under these conditions, fluorescent signals derived from the GPC-specific mAbs and the neutralizing VHHs showed some co-localization with calculated Pearson’s correlation coefficient values below 0.2 (Fig. 3a,d). By contrast, fluorescent signals revealed by the neutralizing VHHs 1.2, 1.69 and 2.96, the non-neutralizing VHH 2.3 and the N-specific mAbs overlapped almost completely, as supported by the deduced Pearson’s correlation coefficient values, which ranged from 0.8 to 0.92 (Fig. 3b,d). Mock infected cells did not show any positive signal when incubated with VHHs and N-specific mAbs (Fig. 3c). These findings suggest that the VHHs bind to N or to a viral epitope that is very close to N.

**Figure 4:**
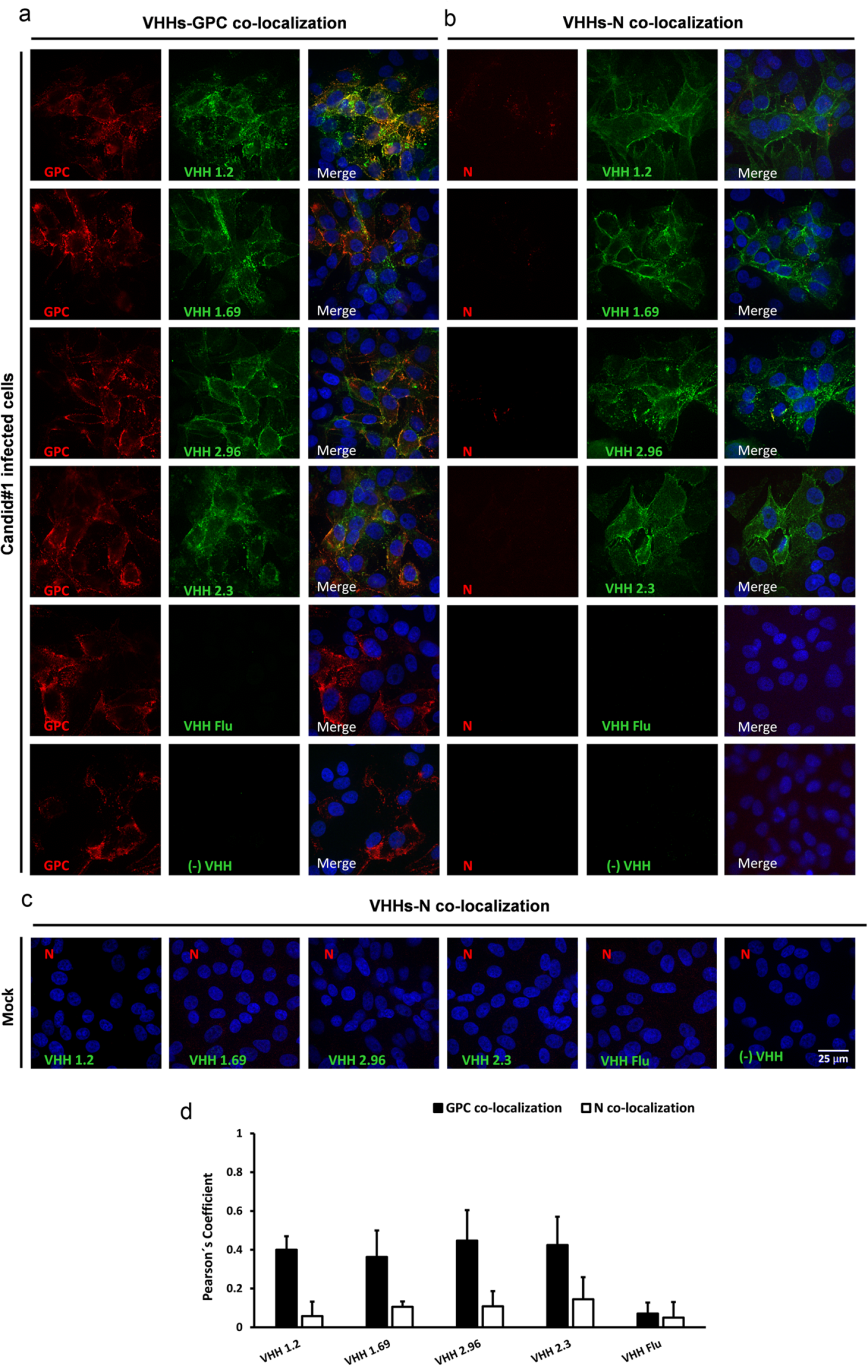
VHHs 1.2, 1.69, 2.96 and 2.3 do not colocalize with JUNV N- or GPC-specific Mabs in fixed non-permeabilized Candid#1 infected cells. Vero cells were mock infected or infected with 150 PFU of Candid#1. At 48 h p.i. cells were first incubated with 10 µM of VHHs followed by incubation with a 1:600 dilution of GPC- (**a**) or N-specific mAbs (**b**) in a CO_2_ incubator. Afterwards the cells were fixed with 2% PFA (without a permeabilization step) and further incubated with an anti-His antibody followed by incubation with the respective secondary antibodies. Anti-His antibody without the presence of VHH was used (−VHH), incubation with a VHH against Flu (VHH Flu) and mock infected cells were used as negative controls. (**c**) Immune-staining was performed in mock-infected cells with VHHs followed by incubation with N-specific mAbs. (**d**) Colocalization of the VHH-specific fluorescent signal with that of the GPC- an N-specific mAbs was determined in 50 cells that showed a positive signal with GPC- or N-specific mAbs. The Pearson’s correlation coefficient (P) was calculated using the Coste’s algorithm provided in the Volocity software package. The scale bar represents 25 µm.

Next, immuno-stainings of Candid#1-infected cells were repeated but now VHHs and the GPC- or N-specific mAbs were allowed to bind to the cells before fixation (*i*.*e*. the permeabilization step was omitted). As expected, under these conditions the GPC-specific mAbs bound to infected cells, indicating that they could recognize viral antigens exposed on the cell surface (Fig. 4a). By contrast, the N-specific mAbs failed to bind to infected cells, which is in line with the assumed cytoplasmic localization expected for N (Fig. 4b). Surprisingly, we found that VHHs 1.2, 1.69, 2.96 and 2.3 bound specifically to non-permeabilized Candid#1-infected cells (Fig. 4a,b), while no signal was detected in mock infected cells (Fig. 4c). Furthermore, the three neutralizing VHHs, as well as VHH 2.3, partially co-distributed with the GPC-specific mAbs under these conditions, with a Pearson´s coefficient value of approximately 0.4 (Fig. 4a,d). Combined with the immuno-fluorescence results obtained from permeabilized infected cells, these data suggest that the Candid#1-specific VHHs 1.2, 1.69, 2.96 and 2.3 are directed against a viral antigen that is accessible on the surface of infected cells.

**Figure 5:**
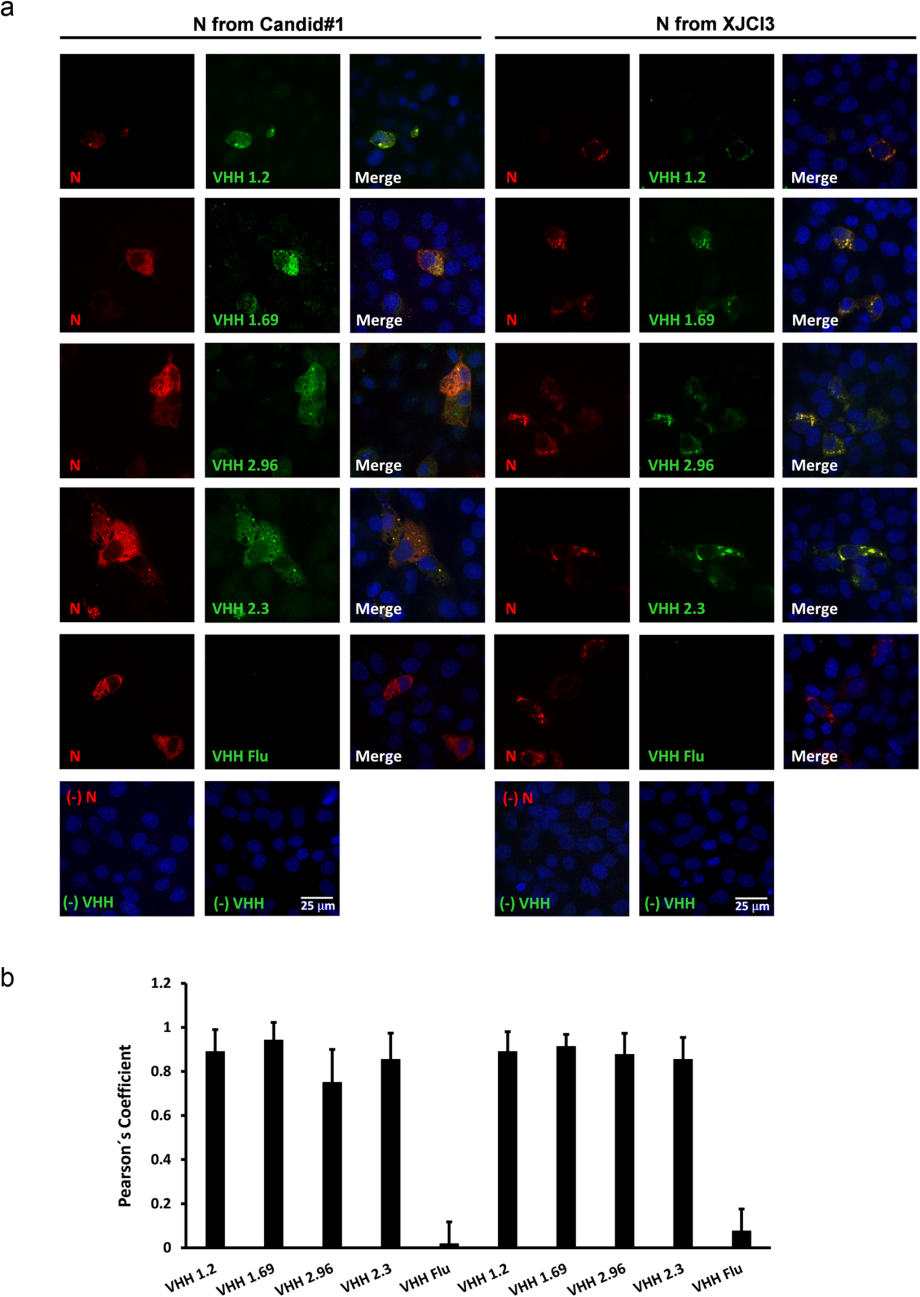
VHHs 1.2, 1.69, 2.96 and 2.3 recognize N derived from Candid# and XJCl3 strains. (**a**) Vero cells at 60% confluence were transfected with 200 ng of expression plasmids coding for N from Candid# (left) or XJCl3 (right) strains. At 48 h post-transfection, the cells were fixed, permeabilized and further incubated with 10 µM of VHHs followed by incubation with N-specific mAbs. Afterwards, cells were incubated with an anti-His antibody and finally incubated with the respective secondary antibodies. Immuno-staining without VHH (−VHH), without N-specific mAb (−N), with a VHH against Flu (VHH Flu) and with GPC-specific mAbs (GPC) were included as controls. Pictures are representative of 3 independent experiments. (**b**) Colocalization of the fluorescent signal derived from immuno-staining with the VHHs was determined in 30 cells that showed a positive signal with N-specific mAbs. The Pearson’s correlation coefficient (P) was calculated using the Coste’s algorithm provided in the Volocity software package. The scale bar represents 25 µm.

Next, we evaluated the binding of the neutralizing VHHs to N-expressing cells. An expression plasmid for N derived from Candid#1 or XJCl3 virus was transiently transfected in Vero cells. Afterwards the cells were fixed, permeabilized and further probed with N-specific mAbs and the VHHs. This revealed that fluorescent signals detected after staining with N-specific mAbs and with VHHs 1.2, 1.69, 1.96 and 2.3 almost completely overlapped (Fig. 5a,b). To note, without prior permeabilization, no signal was observed for the N-specific mAbs in N-transfected cells (Supplementary Fig. 3a). None of the VHHs could stain cells that had been transfected with a GPC expression vector that either contained GPC from Candid#1 or XJ strains (Supplementary Fig. 3b). Collectively these results point out that the viral target of the neutralizing VHHs is a part of the N protein that is exposed on the cell membrane. Notably, cell surface accessibility of this part of the N protein occurs in the context of a viral infection, but not when N is expressed alone.

**Figure 6:**
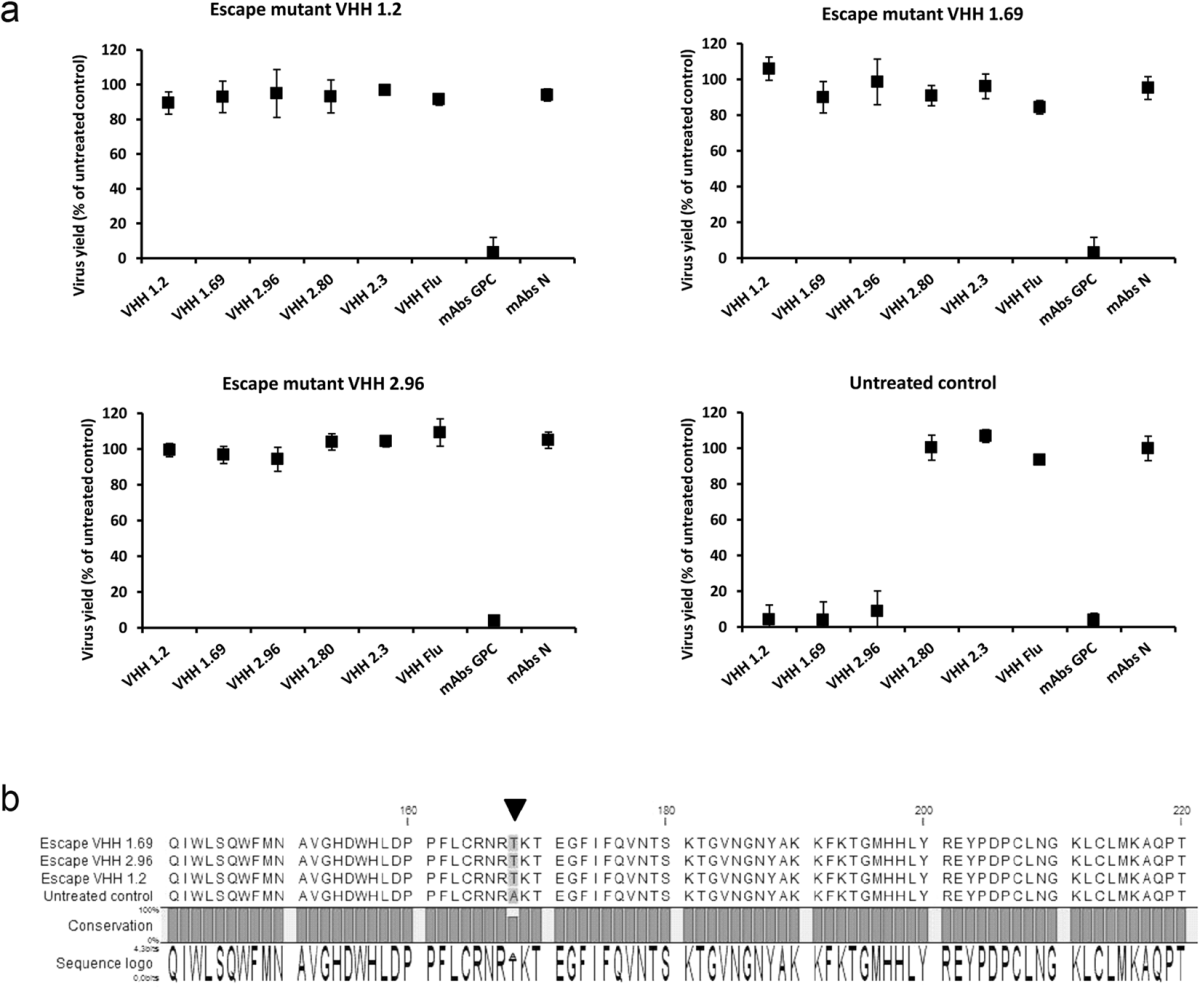
VHHs 1.2, 1.69 and 2.96 Candid#1 escape viruses carry a point mutation in GPC. *In vitro* escape mutants were generated by 3 passages of Candid#1 in the presence of 1000-fold the IC_50_ of the VHHs. (**a**) After the passages, escape viruses were evaluated in a neutralization assay by using 50 PFU of the respective escape viruses and 1 µM of VHHs or GPC- and N-specific mAbs. Neutralization was determined by ELISA. Results are expressed as percentage relative to the untreated control. The mean of three independent experiments (n = 3) is shown, where the error bars represent the standard deviation of the mean (SD). (**b**) Multiple alignment of the predicted amino acid sequence of part of the GPC from the escape mutants obtained under selective pressure with the neutralizing VHHs is shown from residues 141 to 220. The black triangle indicates the altered position in GPC of the escape viruses.

**Figure 7:**
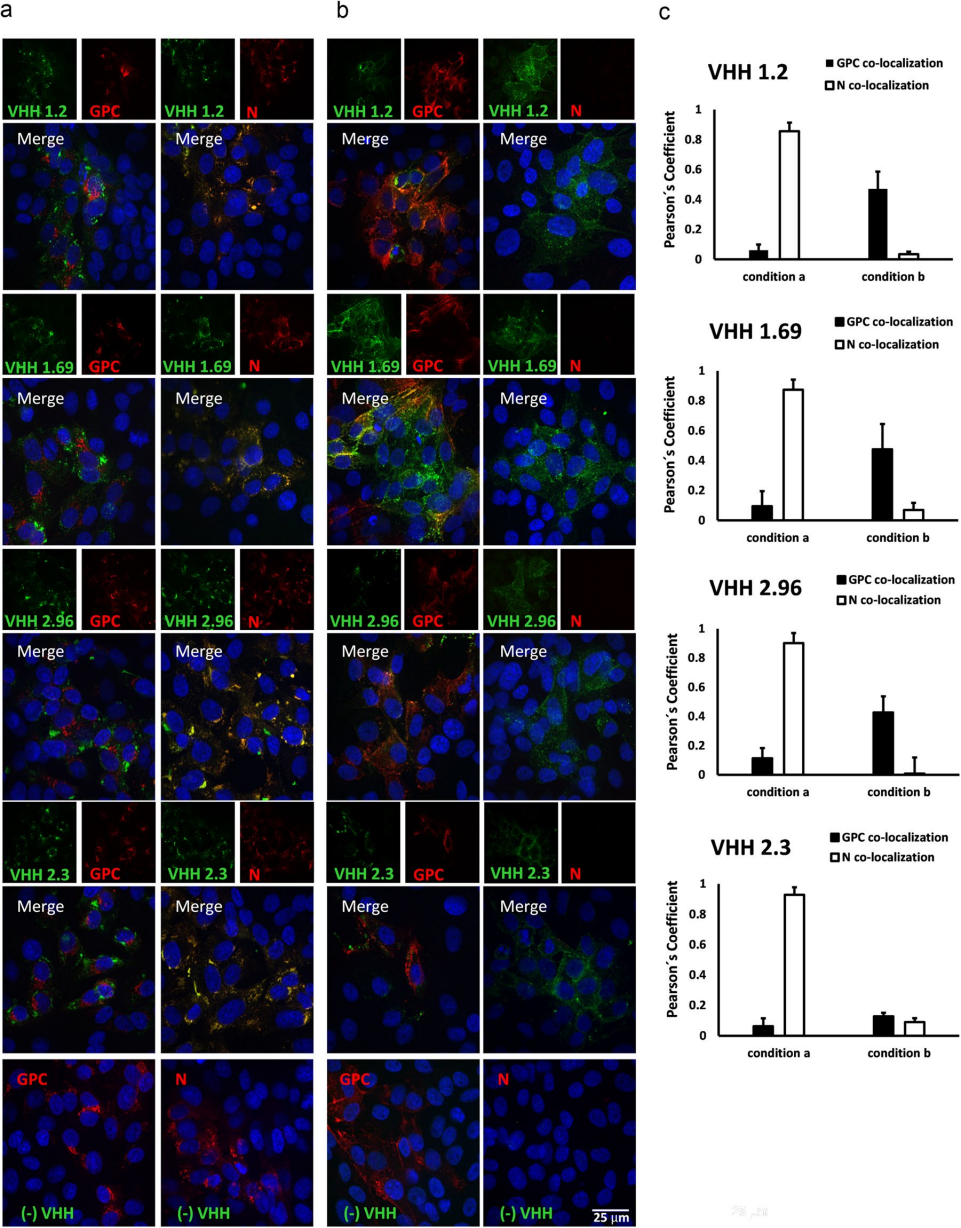
VHHs 1.2, 1.69 and 2.96 colocalize with N of the VHHs escape mutant viruses in infected cells. Vero cells were mock infected or infected with 150 PFU of VHHs escape mutant viruses obtained after selection with VHH1.2, VHH 1.69 or VHH 2.96, respectively. At 48 h p.i. the cells were processed for IFA either before fixation and without permeabilization (**a**) or after fixation followed by permeabilization (**b**). Monolayers were incubated with 10 µM of the indicated VHHs followed by incubation with GPC- or N-specific mAbs. Afterwards, the cells were incubated with an anti-His antibody and finally incubated with the respective secondary antibodies. Control staining in the absence of VHH (−VHH) were included (lower panels). Representative results are shown for the VHH 1.69 escape mutant virus. Similar results were obtained for the VHH 1.2 and VHH 2.96 escape mutants (pictures not shown). (**c**) Colocalization of the fluorescent signal derived from immuno-staining with the VHHs was determined in 50 cells that showed a positive signal with GPC- or N-specific mAbs for each condition. The Pearson’s correlation coefficient (P) was calculated using the Coste’s algorithm provided in the Volocity software package. The scale bar represents 25 µm.

Selection of *in vitro* neutralization escape viruses followed by nucleotide sequence analysis is a conventional method to study the potential binding site of a virus neutralizing antibody or VHH^26^. Therefore, we passaged Candid#1 virus in the presence of VHH 1.2, 1.69 or 2.96 and the resulting escape viruses were characterized. Candid#1 virus was also passaged in the presence of non-neutralizing control VHHs 2.3, VHH 2.80 and an irrelevant VHH directed against an influenza A virus protein. No viruses that had acquired resistance against VHH 1.2, 1.69 or 2.96 were observed when Candid#1 virus was passaged in the presence of the control VHHs (Supplementary Fig. 4). However, Candid#1 escape viruses obtained with any of the three neutralizing VHHs were also resistant to neutralization by the two other VHHs but remained susceptible to neutralization by GPC-specific mAbs (Fig. 6a). Surprisingly, no mutation was detected in either the N (the likely target of the neutralizing VHHs based on the immune-fluorescence analysis) or Z genes of these VHH escape viruses. Instead, we found an alanine to threonine amino acid substitution at position 168 in the predicted GPC sequence (Fig. 6b). To evaluate if the loss of neutralization was due to the inability of the VHHs to bind to the escape mutant viruses, immunofluorescence assays were performed. In line with the unaltered N sequence, all three neutralizing VHHs could still bind to cells that had been infected with the escape mutant viruses. N-specific mAbs and the VHHs co-localized when cells had been fixed and permeabilized prior to immune-staining (Fig. 7a,c). VHHs 1.2, 1.69 and 2.96 also still partially co-localized with GPC when cells were immuno-stained prior to fixation (Fig. 7b,c). These results indicate that, although resistant to the VHH neutralization, the N protein of VHH escape mutants displays the same antigenicity as the parental Candid#1 virus and that the N protein could be readily detected on the surface of non-permeabilized cells that had been infected with the escape viruses.

### Candid#1-neutralizing VHHs do not neutralize pathogenic JUNV

**Figure 8:**
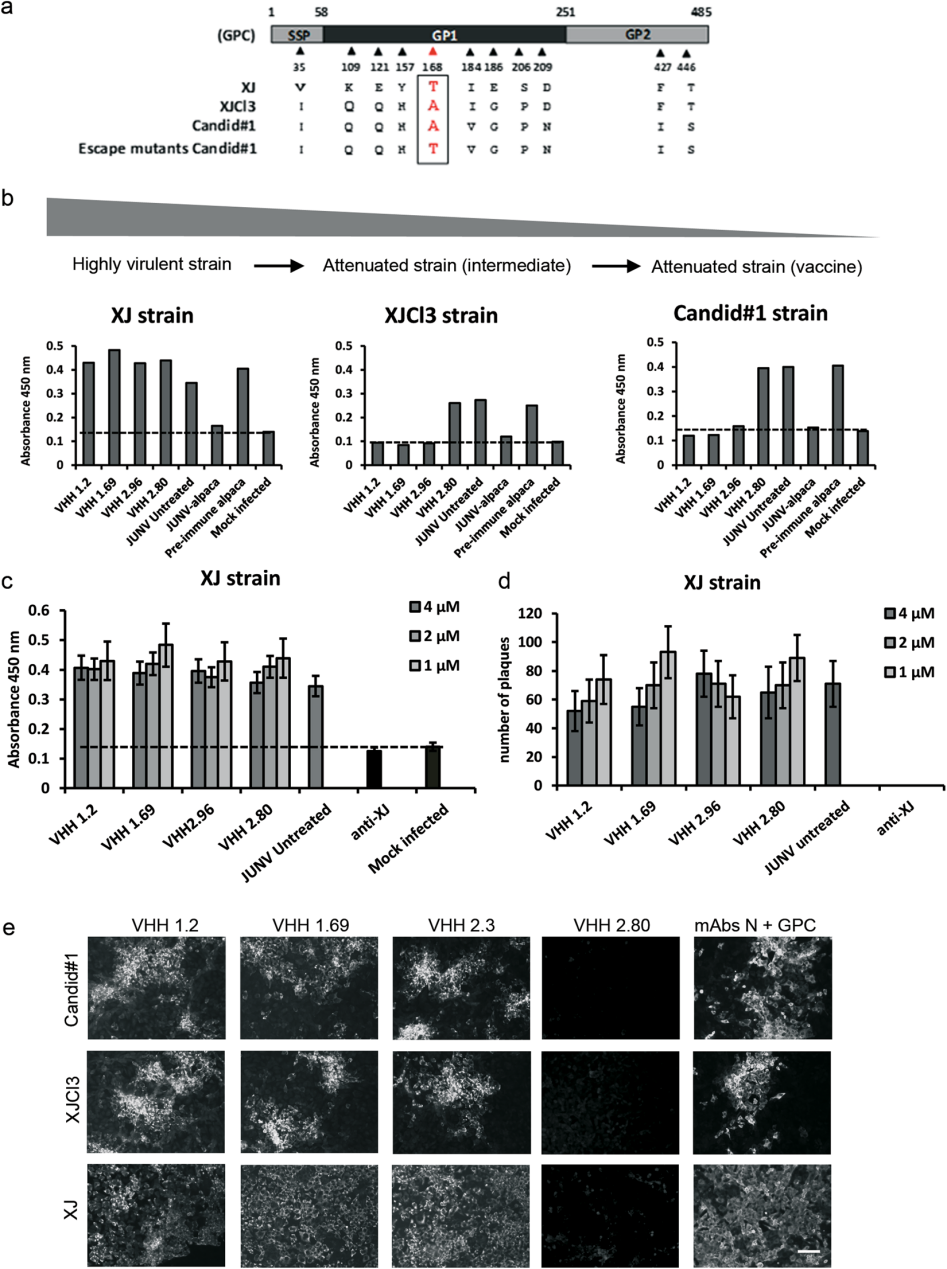
VHHs 1.2, 1.69 and 2.96 bind to cells infected with the pathogenic strain XJ but do not neutralize this virus. (**a**) Amino acid sequence alignment of part of GPC from JUNV XJ, XJCI3 and Candid#1 (adapted from Droniou-Bonzom *et al*.^39^). The last line of the alignment represents part of the sequence of GPC from the VHHs escape mutants that were obtained during this work. The mutation at position 168 is shown in red. (**b**) XJ, XJCl3 or Candid#1 (50 PFU) were incubated with 1 µM of each VHH and then added to Vero cells that had been seeded in 96 well-plates. The pre-immune and Candid#1-immune alpaca serum were evaluated at 1/50 dilution. At 72 h p.i., cells were fixed and virus infection was quantified by ELISA. The scheme at the top represents the path to attenuation of the highly pathogenic XJ strain, over XJCI3 to Candid#1. (**c**) 50 PFU of XJ strain were incubated with different concentration of the VHHs or with 1/50 XJ-immune guinea pig serum and then added to Vero cells seeded in 96-well plates. At 3 days p.i. cell monolayers were fixed and virus infection was quantified by ELISA. (**d**) XJ strain virus (100 PFU) was incubated with different concentrations of VHHs or with 1/50 XJ-immune guinea pig serum (anti-XJ) and then added to Vero cells seeded in 24 well-plates. At 7 days p.i. the cell monolayers were fixed and viral plaques were determined by counter staining of the remaining cells with crystal violet. Results in (c) and (d) represent the mean of three independent experiments (n = 3) and error bars represent the standard deviation of the mean. (**e**) Vero cells growth in coverslips at 90% confluence were infected with 150 PFU of Candid#1, XJCl3 or XJ strains. At 48 h p.i. the cells were fixed, permeabilized and afterwards incubated with 10 µM of the indicated VHHs or a mix of N- and GPC-specific mAbs. Monolayers were further incubated with an anti-His antibody to detect the VHHs and finally the corresponding secondary antibodies were added. The coverslips were mounted and visualized by immunofluorescence. Scale bar represents 100 µm.

Candid#1 is an attenuated JUNV strain that was derived from the pathogenic XJ strain by serial passaging in guinea pigs, mouse brain, and cultured fetal rhesus monkey lung cells^34^. The immunogenicity and protective efficacy of Candid#1 used as a live attenuated vaccine has been tested in animal models and in clinical trials^35–37^. The surmised mechanism of protection of Candid#1 vaccine-mediated immunity is the suppression of virus entry by neutralizing antibodies that can be induced by this vaccine. Interestingly, the A168T variant obtained by selection of *in vitro* Candid#1 VHH neutralization escape viruses recreates the N-linked glycosylation site that is found in the pathogenic XJ isolate of JUNV that was lost in the attenuation process that resulted in Candid#1^20,38,39^ (Fig. 8a). Based on this, it was expected that Candid#1 neutralizing VHHs would fail to inhibit the pathogenic XJ strain of JUNV. A modified neutralization assay, in which virus replication was measured by ELISA, showed that VHHs 1.2, 1.69 and 2.96 neutralized the attenuated XJCl3 and Candid#1 viruses equally well (Fig. 8b). However, *in vitro* replication of the pathogenic XJ strain was not prevented by any of the three Candid#1-neutralizing VHHs, not even when these were applied at up to 4 µM concentration (Fig. 8b,c). Similar results were obtained when a plaque assay was used as a readout (Fig. 8d). To note, the hyperimmune plasma of the Candid#1 immunized alpaca (JUNV-alpaca) could inhibit XJ replication *in vitro* (Fig. 8b) indicating the presence of neutralizing antibodies, conventional or heavy chain-only antibodies, other than the VHHs explored in this work, which were obtained by probing a phage display library derived from the immunized alpaca. Despite the lack of inhibition, the Candid#1 neutralizing VHHs did bind to XJ-infected cells as evidenced by immunofluorescence microscopy (Fig. 8e). These results are in agreement with the observation that the Candid#1 neutralizing VHHs could still specifically bind to cells that had been infected with the escape viruses. Therefore, although amino acid residue 168 in the GPC sequence influences the neutralizing activity of the VHHs 1.2, 1.69 and 2.96 against the JUNV strains, the fact that an XJ-associated determinant is recognized by these VHHs in XJ-infected cells points out that another protein, presumably N, is the target of these Candid#1-neutralizing VHHs.

## Discussion

The aim of this project was to obtain single domain antibodies against JUNV with the purpose to explore this technology as a potential therapeutic option for AHF. The main conclusion of our work, however, is that VHHs that are most likely directed against the N protein of JUNV can neutralize the Candid# virus. These VHHs were able to target N at the plasma membrane of infected cells and prevent viral replication by disturbing virus entry. This is unexpected, because there is no evidence reported yet that the N protein of Arenaviruses is involved in these early steps of the virus life cycle. The three neutralizing VHHs were obtained after immunization of an alpaca with UV-inactivated Candid#1, an attenuated strain of JUNV. These VHHs, named 1.2, 1.69 and 2.96, showed binding activity for both attenuated and pathogenic JUNV strains, but only exhibited a potent *in vitro* inhibitory activity against the attenuated strains Candid#1 and XJCl3. Besides, a membrane-binding pattern observed in Candid#1 infected cells, the three VHHs did not or at best partially co-localize with GPC-specific mAbs and failed to bind to GPC-expressing cells. In addition, the three neutralizing VHHs bound to permeabilized transfected cells that expressed N from Candid#1 or XJCl3 and colocalized with N-specific mAbs in Candid#1 infected cells. Unexpectedly *in vitro* selected Candid#1 mutant viruses that escaped the neutralizing activity of VHHs 1.2, 1.69 and 2.96 had wild type N and Z proteins, and an A168T mutation in the GP1 domain of GPC. GPC is posttranslationally cleaved into GP1, GP2 and a stable signal peptide (SSP) which remains associated with the processed protein to form the mature, trimeric GPC. GP1 is the virion attachment protein and is associated with the membrane anchored GP2 which in turn interacts with SSP. In line with an unaltered N sequence, the three VHHs could still bind to cells that had been infected with the escape variant viruses. Our findings thus show that VHHs that bind to JUNV N can prevent Candid#1 virus entry and syncytium formation (membrane fusion), *i*.*e*. 2 processes that depend on GPC.

These striking properties of these JUNV N-specific VHHs may be ascribed to their particular structure. VHHs consist of a single variable antibody domain with a molecular weight of 12–15 kDa. Their small size, ease of production, high solubility, compact conformation, low tendency to form aggregates and high tissue penetration capacity make VHHs promising therapeutics^40,41^. Indeed, several Nanobodies® are being evaluated in clinical trials. One of the most advanced is ALX-0171 developed by Ablynx Inc. to treat disease caused by human respiratory syncytial virus (RSV) infection. This trivalent monospecific VHH targets the RSV fusion protein (F) and its use was associated with a reduction of viral replication and symptoms in RSV-infected children^42^. We have recently reported on two potent RSV neutralizing VHHs that selectively bind to the prefusion form of RSV F^29^. Furthermore, repeated daily oral administration of a VHH against rotavirus, delivered as part of a crude yeast extract, to male children with acute watery diarrhea was associated with a modest therapeutic effect^43,44^.

*In vitro*, antibodies can inhibit viral replication by different mechanisms: the induction of virion aggregation, destabilization of the virion structure, inhibition of virion attachment to target cells, inhibition of the fusion of the virion lipid membrane with the host cell membrane, prevention of viral genome release, inhibition of a function of the virion core through a signal transduced by an antibody, intracellular effects exerted by transcytosing IgA, and binding to nascent virions that blocks their budding or release from the cell surface^45^. The neutralizing VHHs described here prevent virion entry. Unexpectedly, neutralizing JUNV VHHs likely target the Candid#1N protein, which hitherto was not known to be involved in JUNV entry. Membrane fusion requires the participation of the viral glycoprotein processing products, GP2 and the cleaved SSP^46,47^. The neutralizing VHHs bound to the surface of intact (non-permeabilized) Candid#1-infected cells, which indicates that N is accessible on the cell surface. The fact that N, assumed to be an internal viral antigen, may be exposed on the cell surface although it lacks any conventional transmembrane domain or canonical targeting motif, may not be totally unexpected. Membrane expression of proteins that lack those motifs have been reported^48,49^. Moreover, a pioneering study of the prototype *Arenaviridae* family member lymphocytic choriomeningitis virus (LCMV) reported on the accessibility of N on the plasma membrane surface of LCMV-infected cells and on the surface of the virions for LCMV N-specific mAbs^50,51^. Although, these LCMV N-specific mAbs and the JUNV N-specific mAbs used in our study completely lack virus neutralization activity *in vitro*, the N-specific VHHs described here potently neutralize the Candid#1 virus. Further studies showed that N-mAbs that recognize epitopes of LCMV NP present on the cell surface of LCMV infected cells, are able to enhance *in vivo* virus clearance^52^. Based on our results, during JUNV infection N epitopes also become exposed on the membrane of infected cells, and become accessible for N-specific VHHs but not for the conventional monoclonal antibodies used in our study.

Our findings are somewhat reminiscent of earlier reports on VHHs that are directed against the VP6 inner capsid protein of rotavirus that yet have neutralizing activity. Conventional antibodies against VP6 do not or very poorly neutralize rotavirus *in vitro*, presumably because the antigen is poorly accessible. However, VP6-specific VHHs can neutralize rotavirus possibly because they can access epitopes on VP6 that are important for viral entry: VP6 is involved in rotavirus entry by binding to the host factor hsp70^53,54^. However, Candid#1N is not known to be involved in virus entry. We note that binding of VHHs 1.2, 1.69 and 2.96 to the surface of non-permeabilized cells was only observed with Candid#1-infected cells but not in cells that overexpressed N. This indicates that N may require an interaction with other viral proteins to reach the plasma membrane. Again some parallel with LCMV may be noted since it has been proposed that a phosphorylated form of the nucleoprotein of this virus interacts with the glycoprotein at the level of the plasma membrane of infected cells and as such N becomes exposed on the cell surface^50,51^. One interesting point to note, that supports the idea of an interaction of Candid#1N with GP1, is the fact that viral escape mutants obtained by selective pressure exerted by the neutralizing VHHs showed one amino acid substitution (A168T) in GP1. However, cells infected with these escape viruses were still recognized by the neutralizing VHHs, indicating that exposure of N at the membrane surface was not affected by the A168T mutation in GP1. The substitution of threonine at position 168 to alanine results in loss of an N-glycosylation site in GP1 that has been acquired during the attenuation linage of JUNV that resulted in Candid#1^20^. Although this change was initially reported to be unrelated to Candid#1 attenuation^38,55^, a recent study related the absence of this N-linked glycosylation motif in GP1 of Candid#1 with an altered processing and reduced surface expression of GP1 in infected cells^56^. A limitation of our study is that we could not yet demonstrate a direct interaction between N and the neutralizing VHHs 1.2, 1.69 and 2.96. Indeed, we have used VHHs 1.2, 1.69 and 2.3 to try to immuno-precipitate N from Candid#1 virus infected cells. However, only VHH 2.3, which is non-neutralizing, was capable of immuno-precipitating N from infected cells (Supplementary Fig. 5). Possibly, the detergent lysis and washing conditions that were used are detrimental for the epitope in JUNV N that is recognized by the neutralizing VHHs described here.

We speculate that neutralization exerted by the JUNV VHHs could be due to a perturbation of the spatial structure of the glycoprotein on the membrane caused by the binding of the VHHs to N (which would be in close proximity to the glycoprotein). It is possible that GP1 from Candid#1 could also require a receptor shift upon acidification in order to promote virus membrane fusion as has been reported for LCMV^57^. Such a receptor shift could perhaps be hindered by VHHs that bind to N. In conclusion, N may be a target of JUNV neutralizing single domain antibodies.

## Methods

### Ethics statement

Immunizations and handling of the alpaca were by an accredited veterinarian according to the local guidelines for animal care and handling, and were authorized by ethical committee for animal experiments of the Vrije Universiteit Brussel (permit No. 13-601-1).

### Virus preparations

A purified stock of Candid#1 strain (kindly provided by Dr. Victor Romanowski, Facultad de Ciencias Exactas, La Plata, Argentina) either concentrated and non-concentrated was prepared as previously described^58^. For alpaca immunization, inactivation of the stock was done by exposing infectious virus to a 30 W UV light at a distance of 10 cm for 8 min.

XJ and XJCl3 virus stocks were routinely maintained at the Laboratorio de Virología, FCEyN, Universidad de Buenos Aires and originally supplied by the Cátedra de Microbiología, Facultad de Medicina, Universidad de Buenos Aires).

### VHH isolation and production

An alpaca (*Vicugna pacos*) was immunized by six weekly intramuscular injections with 50 µg of purified and concentrated UV-inactivated Candid#1 strain of JUNV (corresponding to an original infectivity of 1.10^7^ PFU) in the presence of Gerbu LQ#3000 adjuvant. The immune plasma collected after immunization was tested to evaluate its neutralizing activity against Candid# 1 (Supplementary Fig. 6). Afterwards, a VHH phage display library from the mononuclear cells of the immunized animal was generated in the phagemid vector pHEN4-HA^59^. The resulting phage display library was enriched for Candid#1-binding VHH candidates by two consecutive rounds of panning on immobilized UV-inactivated Candid#1 virions. Ninety-five random clones were selected after the first and 96 random clones after the second panning round. Periplasmic extracts (PE) were prepared from each of these clones by using lysis-phosphate buffer (50 mM Na_2_PO_4_H; 300 mM NaCl). The resulting PE extracts were evaluated by PE-ELISA using coated inactivated Candid#1 virions as target. Thirty Candid#1-binding candidates were obtained after the first and second panning round combined (Supplementary Fig. 1a).The PE extracts were then evaluated in a Candid#1 neutralization assay with a criteria to define the potential neutralizing candidates as those PEs with a neutralization activity higher than 50% compared with a PE obtained from control bacteria. Based on these criteria we selected 9 VHH candidates from the first and two from the second panning round (Supplementary Fig. 1b). The nucleotide sequence of thirty Candid#1-binding candidates was determined and clustered phylogenetically using a Maximum Likelihood method (Supplementary Fig. 2a).

The VHH coding sequences that were determined based on the PE-ELISA selection were transferred into the pHEN6-6x His tag expression plasmid. Briefly, VHHs coding sequence from pHEN4-HA were amplified by PCR with the Primer A6E 5′GATGTGCAGCTGCAGGAGTCTGGRGGAGG 3′ and Primer 38 5′GGACTAGTGCGGCCGCTGGAGACGGTGACCTGGGT 3′containing a PstI and BstEII restriction site (underlined), respectively. The PCR products were then digested with the respective enzymes and sub-cloned in the pHEN6-6x His tag vector in frame with a C-terminal His-6 tag.

Following transformation of *E*. *coli* cells with the pHEN6-VHH plasmids, single colonies were used to inoculate 5 ml LB pre-cultures. After overnight incubation, 1 ml of pre-culture was used to inoculate 300 ml LB and further induced with 1 mM IPTG. PE were prepared by using lysis-phosphate buffer and the VHHs were purified by Ni-NTA chromatography.

### Plasmids expressing viral proteins

To construct plasmids expressing the viral proteins, total RNA was extracted from Candid#1 or XJCl3 infected cells and the cDNA was further synthesized by using the arena primer (ARS1) as described elsewhere^58^. The Junin mammarenavirus strain XJCl3 nucleoprotein and glycoprotein precursor gene sequences have been deposited in Genbank under accession numbers MG189700.1 and MG189701.1, respectively. The coding sequences for the viral proteins N and GPC, were PCR amplified using primers compatible with the Gateway recombination system. The following primers were used: Gate N Cand#1 fw 5′GGGGACAACTTTGTACAAAAAAGTTGGCATGGCACACTCCAAAGAGG-3′, Gate N Cand#1 rv 5′-GGGGACAACTTTGTACAAGAAAGTTGGCAACAGTGCATAGGCTGCCTTCG-3′, Gate GPC fw 5′-GGGGACAACTTTGTACAAAAAAGTTGGCATGGGGCAGTTCATTAGC-3′, Gate GPC rv 5′-GGGGACAACTTTGTACAAGAAAGTTGGCAATTAGCATTTCAGAAATTAAGA AGTC-3′. The plasmid pSEL1 + 2L was used as the destination vector.

Transfection was performed in Vero cells by using Fugene following the manufacturer´s instructions. Briefly, 20,000 cells were seeded in each well of a 96-well plate, and 200 ng of plasmid was incubated with 0,3 µl of Fugene in Optimem in a final volume of 10 µl during 15 min. Afterwards the mix was added to the cells that had been refreshed with 100 μl of Optimem without serum and incubated at 37 °C in a CO_2_ (5%) incubator. At 48 h post transfection the cells were processed according to the respective experiment.

### Enzyme-linked immunosorbent assays (ELISAs)

PEs were prepared from single colonies of *E*. *coli* that were retained after panning on immobilized UV-inactivated Candid#1 virions. Wells of a 96-well plate were coated with 1.7 µg/µl of concentrated UV-inactivated Candid#1 virus that was diluted in carbonate/bicarbonate buffer overnight at 4 °C. Wells coated with the coating buffer alone were used as negative control. The wells were washed three times with PBS containing 0.1% Tween (PBST) and blocked with 1% casein in PBS for 1 h at room temperature. After blocking, 100 µl of PE was added per well and incubated 1 h at room temperature. Wells were then washed with PBST and further incubated with a mouse anti-HA antibody followed by an anti-mouse antibody conjugated to HRP. After the addition of the HRP substrate, the optical density was measured at the wavelength of 450 nm and a reference wavelength of 650 nm.

To determine binding of the purified VHHs to Candid#1 infected cells, Vero cells were seeded in 96-well plates at 20,000 cells per well and after overnight incubation at 37 °C were mock infected or infected with 50 PFU of Candid#1. Seventy two hours post infection (p.i.) cells were fixed with 2% paraformaldehyde (PFA) and then permeabilized with 0.15% Triton X-100 in PBS. After permeabilization the cells were blocked for 1 h at room temperature with 3% BSA 0.15% Tx-100 in PBS and incubated afterwards for 2 h with 1/10 serial dilution of the VHHs. Subsequently, the plates were incubated for 1 h at room temperature with a mouse anti-His antibody followed by incubation with anti-mouse antibody conjugated to HRP. After the addition of the HRP substrate optical densities were determined in each well as described above.

VHH-ELISA was also performed by incubating infected or mock infected Vero cells with VHHs before fixation. In that case, the cells were first incubated with 1/10 serial dilutions of VHHs in Optimem® medium for 2 h in a 4% CO_2_ incubator at 37 °C. After incubation the cells were washed three times with PBS, fixed with 2% PFA and subsequently blocked and processed as mentioned above (without the permeabilization step).

### Virus neutralization assays

Candid#1 virus (150 PFU) was mixed with 100 µl of PE that had been incubated for 15 min at 65 °C to heat kill residual *E*. *coli* cells, and incubated at 37 °C during 1 h. Subsequently, the virus/PE mixture was added to 90% confluent Vero cells that had been seeded in 24 well plates. After 1 h incubation at 37 °C in a CO_2_ (5%) incubator, the inoculum was removed, cells were washed three times with medium and replenished with 0.5 ml of semisolid medium containing 0.7% methylcellulose in 0.5% FCS DMEM. At 7 days p.i. cells monolayers were fixed with 4% PFA and further stained with crystal violet to count the viral plaques.

To determine the virus neutralizing activity of the purified VHHs, Candid#1 (50 PFU) was mixed with 1/10 serial dilutions of VHHs in Optimem® without serum and incubated at 37 °C during 1 h in a water bath. Subsequently the mix virus/VHH was added to 90% confluent Vero cells seeded in 96 well plates and incubated 1 h in a 4% CO_2_ incubator at 37 °C. After infection, the cells were washed three times with medium and replenished with fresh medium without serum. Seventy two h p.i. the cell monolayers were washed with PBS, fixed with 2% PFA and then permeabilized with 0.15% Triton X-100 in PBS. After permeabilization the wells were blocked for 1 h at room temperature with 3% milk in PBS and incubated afterwards with 1:2000 of a mix of N-mAbs and a mix of 1:2000 GPC-mAbs in PBS with 1.5% milk- 0.05% Tween. Finally, the wells were incubated 1 h at room temperature with an anti-mouse antibody conjugated to HRP and viral plaques were visualized and counted after addition of TrueBlue^TM^ peroxidase substrate. Alternatively ELISA peroxidase substrate was used to quantify the amount of viral antigen using an ELISA reader. GPC-mAbs mix was composed of 1:1:1 parts of GB03-BE08, QD04-AF03 and QC03-BF11 GPC-specific monoclonal antibodies (Biodefense and Emerging Infections Research Resources Repository (Bei resources)). N-specific mAbs mix was composed of 1:1:1 parts of SA02-BG12, QB06-AE05 and NA05-AG12 monoclonal antibodies (Bei resources)^60^.

Neutralization assays with purified VHHs were performed in Vero cells by conventional plaque assays, by the incubation of cell monolayers with semisolid medium for 7 days followed by fixation with 4% PFA and further visualization of viral plaques after crystal violet staining.

### Immunofluorescence microscopy

Vero cells seeded in 96-well plates with glass bottoms were mock infected or infected with 50 PFU of Candid#1. Forty eight h p.i. cells were fixed with 2% PFA and then permeabilized with 0.15% Triton X-100 in PBS. After permeabilization the wells were blocked for 1 h at room temperature with 3% BSA 0.15% Tx-100 in PBS and incubated afterwards 2 h with 10 µM of purified VHHs. Subsequently, cells were incubated 1 h at room temperature with a mouse anti-His antibody (1:2000, GE) followed by incubation with anti-mouse antibody conjugated to Alexa 488. Hoechst 1:1000 was added together with the secondary antibody. Monolayers were replenished with PBS and analyzed by confocal microscopy.

Immuno-fluorescence of non-fixed cells was performed by first incubating the cells with 10 µM of purified VHHs in Optimem® without phenol red indicator during 2 h in a 4% CO_2_ incubator at 37 °C. After incubation, the cells were washed three times with PBS, fixed with 2% PFA and subsequently blocked and processed as mentioned above (without permeabilization).

For double labeling (co-localization assays), monolayers were first incubated with 10 µM of VHHs during 2 h and after washing were incubated during 1 h at RT with 1:600 dilution of the mAbs anti-GPC or anti-N. Afterwards monolayers were incubated with a rabbit anti-His antibody (1:600, Thermo Fisher) for 1 h at RT. After washes the conjugated antibodies goat anti-mouse Alexa 555 (1:600) and goat anti-rabbit Alexa 488 (1:600) were added in the presence of 1:1000 of Hoesch and incubated for 1 h at RT.

Colocalization in confocal images was calculated by statistical analysis with Volocity 6.3.1 software (Perkin Elmer, Coventry, UK). The Pearson’s correlation coefficient (P) was calculated using the Coste’s algorithm provided in the Volocity software package. Thirty to fifty cells positive for N- or GPC-specific mAbs were processed per setting.

### Pre and post attachment, post-entry and pre-treatment assay

For the pre-attachment assay 50 PFU of Candid#1 virus were mixed with 1/10 serial dilutions of VHHs in Optimem® without serum and incubated for 1 h in a 37 °C. The mix was added to Vero cells seeded in 96 well plates and incubated 1 h at 37 °C. After infection, cells were washed three times with medium and replenished with fresh medium without serum. For the post-attachment assay 50 PFU of Candid#1 virus were added to pre-chilled Vero cells and incubated for 1 h at 4 °C. Cells were washed three times with cold medium to remove the unbound virus and incubated afterwards with 1/10 serial dilutions of VHHs during 1 h at 37 °C. After incubation the cells were washed three times with medium and replenished with fresh medium without serum.. For the cell pre-treatment assay, Vero cells were incubated with 1/10 serial dilutions of VHHs 1 h at 37 °C, washed 3 times with cold medium and then chilled on ice for 15 min. Cells were then incubated with 50 PFU of Candid#1 virus 1 h at 4 °C and after washes with cold medium were shifted at 37 °C.

For all the conditions, the extent of viral replication was measured at 72 h p.i. by ELISA by using a mix of N- and GPC-specific mAbs as described above.

### Syncytium formation assay

Vero cells seeded in 96 well plates were mock infected or infected with Candid#1 at an MOI of 0.05. At 48 h p.i. 1/10 serial dilutions of VHHs were added to the cells and incubated for 2 h at 37 °C. Cells were then washed with fresh medium and covered with DMEM pH 5.0 during 3 h in order to induce syncytium formation. Subsequently cells were replenished with DMEM pH 7.0 for 1 h and fixed with methanol for 10 min at −20 °C for Giemsa staining. After fixation cells were incubated with 0.4% Giemsa and examined for the presence of multinucleated cells by light microscopy. For IFA, GPC staining was performed as described above.

### Generation of escape mutants

*In vitro* Candid#1 escape viruses were isolated by selection with VHHs. Vero cells were infected with serial dilutions of Candid#1 virus in the presence of 1000-fold the median inhibitory concentration of VHHs obtained from neutralization assay. Escape viruses were selected using standard techniques. After 3 passages in the presence of the VHHs, escape virus was amplified in Vero cells still in the presence of the respective VHHs. Total RNA was extracted from the supernatants and cDNA was synthetized using the arena primer (ARS1) as described elsewhere^58^. The PCR fragment corresponding to the nucleoprotein, glycoprotein precursor and Z protein were analyzed by sequencing after amplification using the following primers: N fw 5′-ATGGCACACTCCAAAGAGG-3′; N rv 5′-CAGTGCATAGGCTGCCTTCG-3′; GPC fw ATGGGGCAGTTCATTAGC-3′; GPC rv 5′-GATGTGTCCTCTTGCGCC-3′; Z fw ATGGGCAACTGCAACGGGGCATC-3′; Z rv 5′-TGGTGGTGGTGCTGTTGGCTCC-3′.

## Electronic supplementary material

Supporting information
