# Supplementary material for: Neutralization of Junín virus by single domain antibodies targeted against the nucleoprotein
Source: Sci Rep. 2018 Jul 30;8:11451. doi: 10.1038/s41598-018-29508-1 (PMC6065417; doi:10.1038/s41598-018-29508-1)
Supplement: Supplementary file 1 — Supporting information [file 41598_2018_29508_MOESM1_ESM.pdf]

Neutralization of Junín virus by single domain antibodies targeted against the nucleoprotein

Florencia Linero[1,2], Claudia Sepúlveda[3], Ioanna Christopoulou[1,2], Paco Hulpiau[2,4], Luis Scolaro[3], and Xavier Saelens[1,2]*

Supporting information

Supplementary figure 1

a

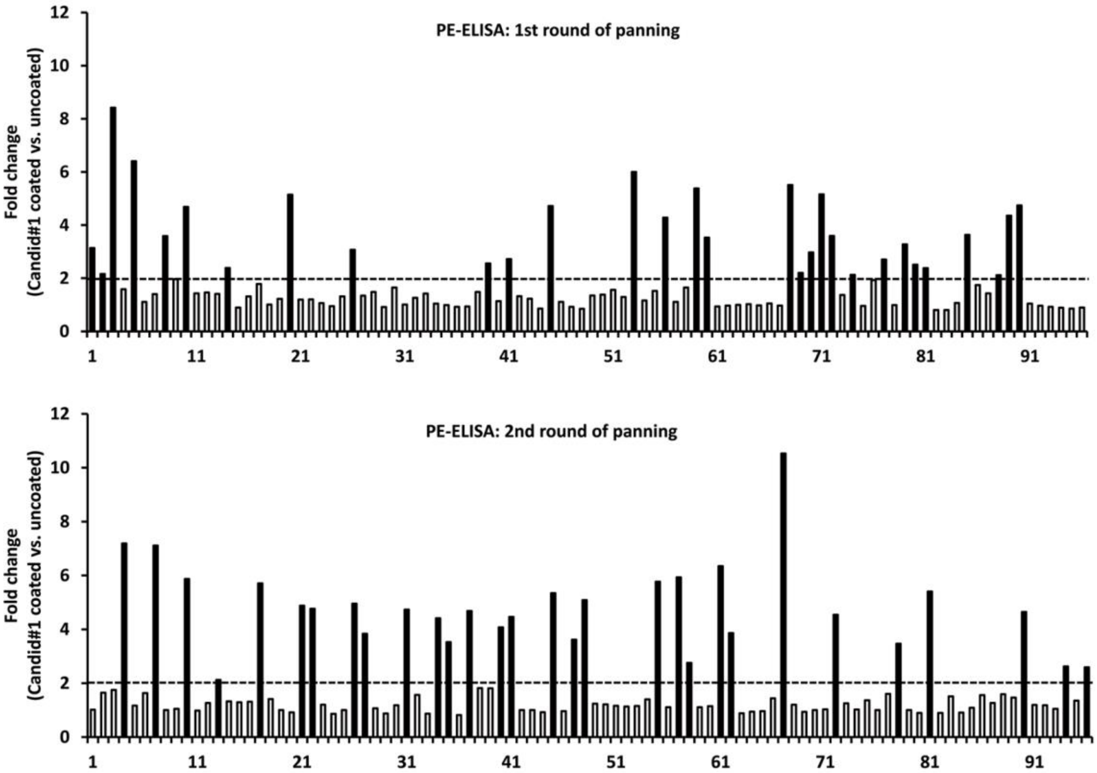

b

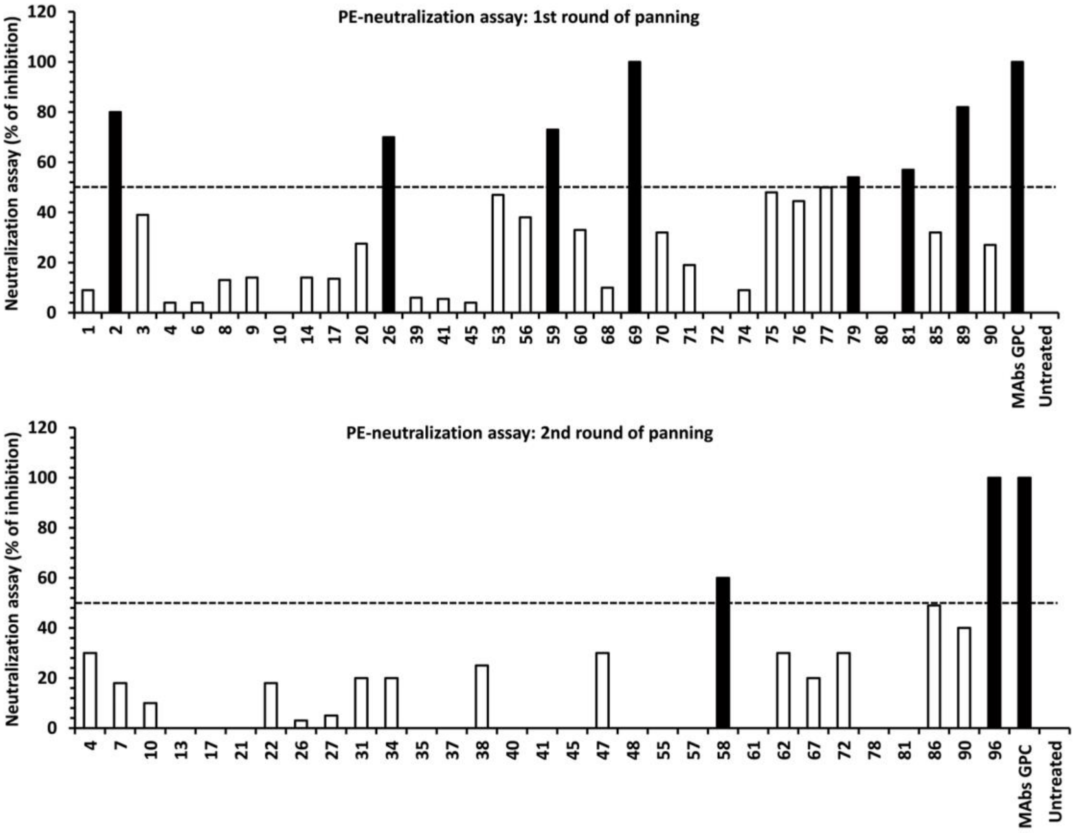

Supplementary figure 1. Biopannig and neutralizing activity of *E. coli* periplasmic extracts (PE) expressing HA-tagged VHHs.

(a) After two round of selection and enrichment in *E. coli*, the PE of 95 and 96 clones per round were screened by a PE-ELISA using a 96 well plate with Candid#1 UV-inactivated coated wells and an anti-HA antibody. The specific Candid#1 binders (black bars) were defined as those with a binding activity measured by ELISA that was two times higher than the uncoated control. (b) The PEs with Candid#1 binding activity were evaluated for their ability to impair *in vitro* virus replication by a conventional neutralization assay. To this end, 50 PFU of Candid#1 virus were incubated with the PEs prior to inoculation of Vero cells. Afterwards the virus-PE mix was added to Vero cells and after 7 days, virus plaques were quantified. A mixture of JUNV-specific neutralizing monoclonal antibodies directed against the virus glycoprotein (MAbs-GPC) was used as a positive control while a PE obtained from uninfected bacteria (untreated) was used as negative control. Neutralization is expressed as percentage of inhibition compared with the untreated control. A 50% inhibition was considered as cutoff for defining the putative neutralizing VHHs (black bars).

Supplementary figure 2

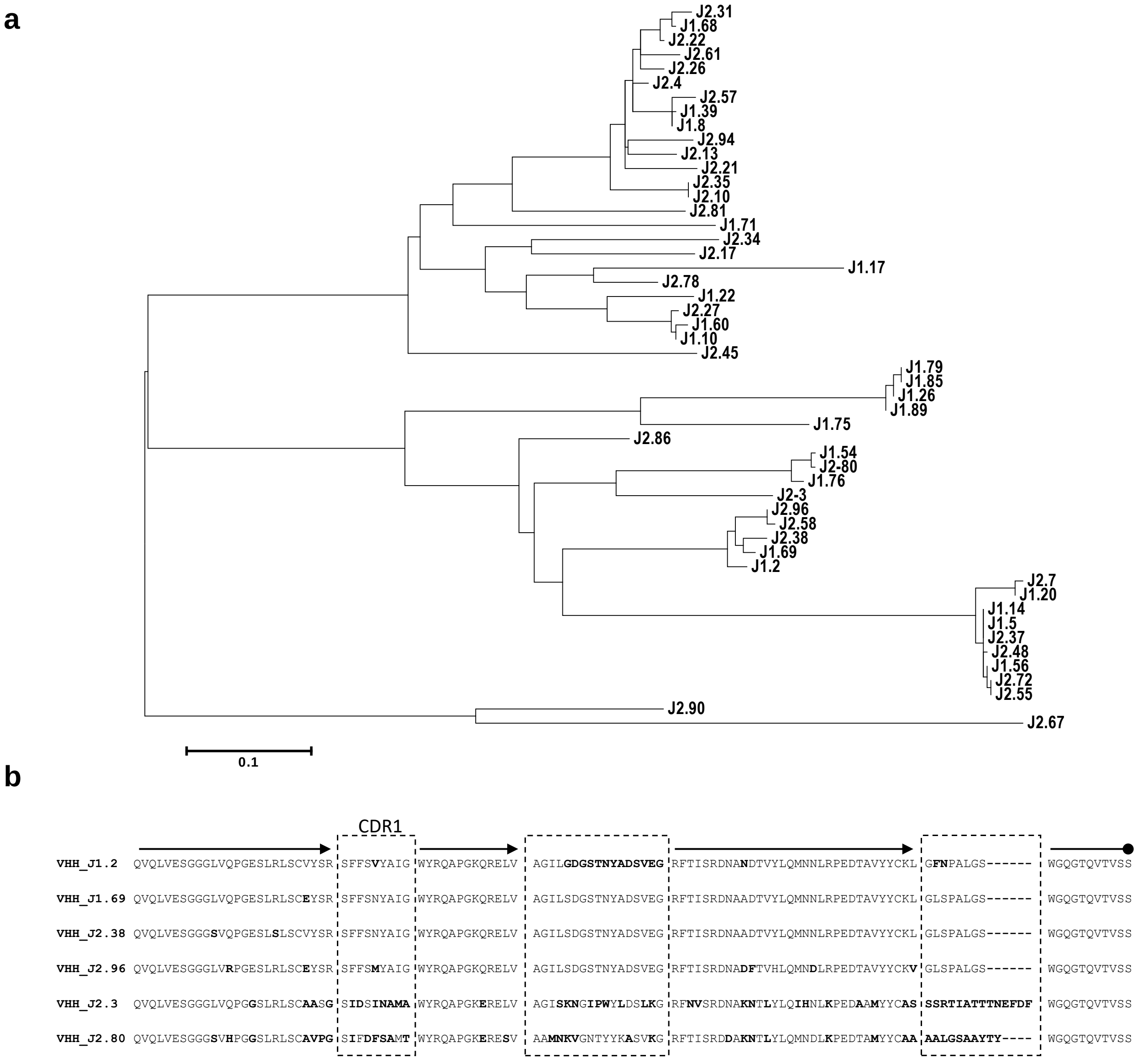

Supplementary figure 2. Molecular Phylogenetic analysis of the Candid#1 binders by Maximum Likelihood method.

(a) The primary sequence homology of the VHHs was inferred by using the Maximum Likelihood method based on Tamura *et al*., 2013 (*Tamura K, Stecher G, Peterson D, Filipski A, Kumar S. MEGA6: Molecular Evolutionary Genetics Analysis version 6.0. Molecular Biology and Evolution. 2013; 30: 2725-2729).* J=Junin virus. First number indicates first (1) or second (2) round of panning and the second number indicates the number of the clone.

(b) Predicted amino acid sequence of the selected VHHs. FR = Framework region; CDR = Complementarity determining region.

Supplementary figure 3

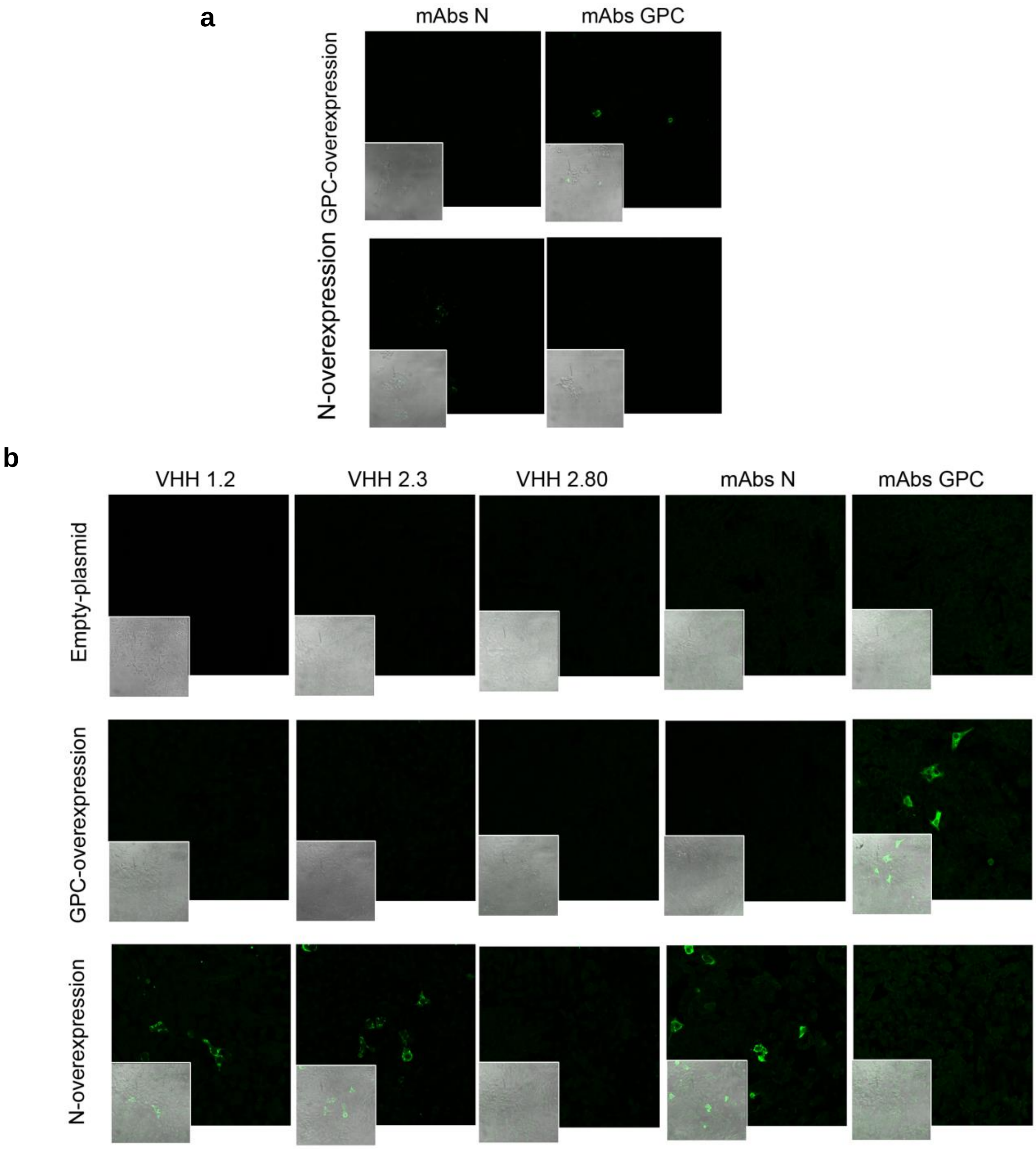

Supplementary figure 3. Immunofluorescence of overexpressing cells of N from XJCl3 and GPC from XJ strains.

Vero cells at 60% confluence were transfected with 200 ng of expression plasmids coding for N from XJCl3 or GPC from XJ strain. (a) At 48 h post-transfection, the cells were fixed (without a permeabilization step) and further incubated with N- or GPC-specific mAbs

followed by incubation with an anti-mouse secondary antibody. (b) Alternatively, at 48 h post-transfection the cells were fixed, permeabilized and further incubated with N- or GPC-specific mAbs followed by incubation with an anti-mouse secondary antibody.

Supplementary figure 4

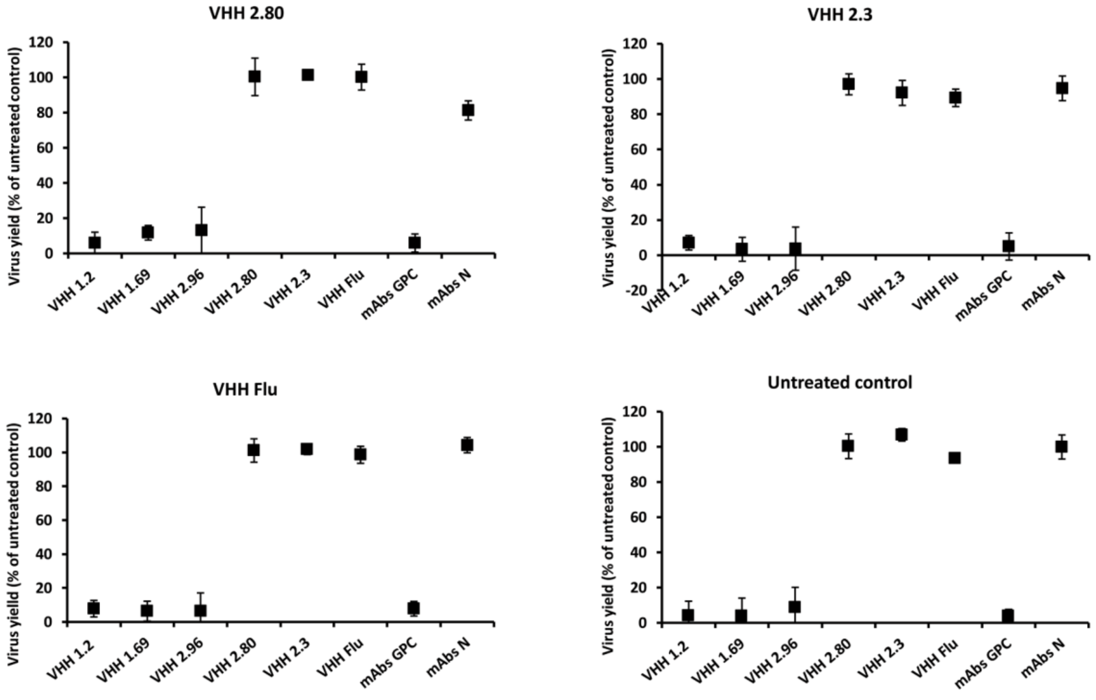

Supplementary figure 4. Neutralization assay of viruses obtained under "selection" by non-neutralizing VHHs.

After 3 passages of Candid#1 in the presence of the VHHs 2.3, 2.80 or VHH Flu at a concentration of 5 µM, viruses were further characterized by neutralization assay using 50 PFU of the respective viruses and 1 µM of VHHs or 1:500 of Mabs. Neutralization was measured by ELISA. Results are expressed as percentage relative to the untreated control. The mean of three independent experiments (n=3) is shown, where the error bars represent the standard deviation of the mean.

Supplementary figure 5

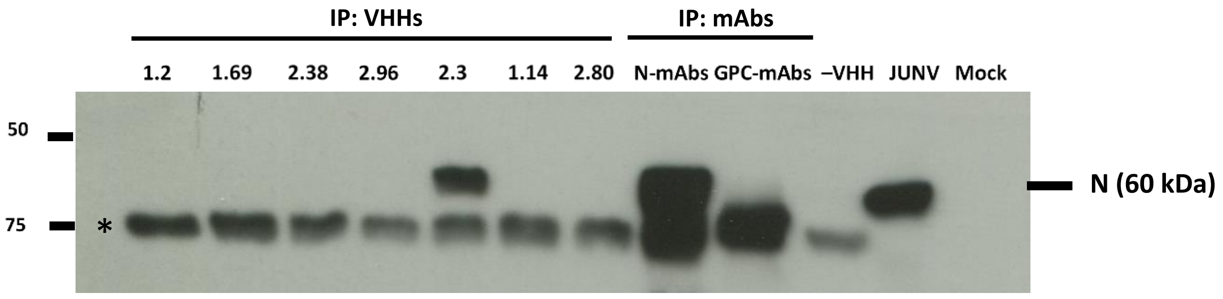

Supplementary figure 5. Immunoprecipitation assay of Candid#1 infected cells with the VHHs.

Vero cells at a confluence of 90% were infected with Candid#1 virus at an MOI of 0.1 PFU/cel. At 72 h p.i. approximately 3 x10^5 cells were resuspended in 200 µl of RIPA buffer per sample. Samples were incubated with the respective VHH at final concentration of 20 µM or with 1/200 of N- or GPC-specific mAbs. The mix was incubated 30 min at RT followed by 90 min at 4°C. Samples containing the VHHs were further incubated in the same condition with 1/100 of an anti-His antibody. A sample that did not contain VHHs (-VHH) was incubated with the anti-His antibody as control. Afterwards 60 ul of protein-A sepharose beads (100 mg/ml) was added to each tube and incubated 30 min at RT followed by 90 min at 4°C. The beads were then washed 3x with RIPA buffer and finally proteins were dissociated by adding 50 µl of denaturing PAGE and boiling. The presence of N was evaluated by Western Blot analysis using a 1:1:1 mixture of N-specific mAbs SA02-BG12, QB06-AE05 and NA05-AG12 (Bei resources). Lysates of Candid#1 and mock infected cells that were not subjected to immunoprecipitation were used as control. * The bands observed at 75 kDa correspond to the mouse Ig chain of either the N-specific monoclonal or anti-His antibodies used for the immunoprecipitation.

Supplementary figure 6

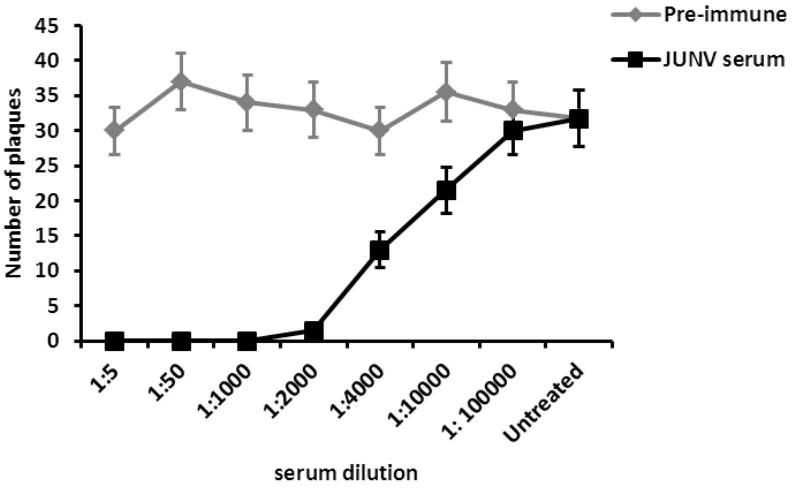

Supplementary figure 6. Neutralizing activity of the serum of the inactivated Candid#1 immunized alpaca.

50 PFU of Candid#1 were incubated with serial dilution of pre-immune serum or Candid#1 immune serum and subsequently used to infect Vero cells. At 7 days p.i. cells were stained with crystal violet and virus plaques were counted. Error bars show the standard variation of the outcome of three independent experiments
